# Testing reliability and validity of the Korean version of Debriefing Assessment for Simulation in Healthcare (K-DASH)

**DOI:** 10.1186/s41077-024-00305-3

**Published:** 2024-08-08

**Authors:** Seon-Yoon Chung, Bu Kyung Park, Myoung Jin Kim, Jenny W. Rudolph, Mary Fey, Robert Simon

**Affiliations:** 1 College of Nursing, University of Wisconsin Oshkosh, 800 Algoma Blvd, Oshkosh, WI 54901 USA; 2 College of Nursing, Kyungpook National University, 640 Gukchaebosang-Ro Jung-Gu, Daegu, 41944 Korea; 3 Mennonite College of Nursing, Illinois State University, Campus, Box 5810, Normal, IL 61790 USA; 4 Center for Medical Simulation, Boston, USA; 5 Massachusetts General Hospital-Institute for Health Professions, Boston, USA; 6 Harvard Medical School, Massachusetts General Hospital, Boston, USA

**Keywords:** Simulation Training, Reliability, Validity, Factor analysis, Debriefing

## Abstract

**Background:**

Use of the Debriefing Assessment for Simulation in Healthcare (DASH^©^) would be beneficial for novice debriefers with less or no formal training in debriefing. However, the DASH translated into Korean and tested for psychometrics is not yet available. Thus, this study was to develop a Korean version of the DASH student version (SV) and test its reliability and validity among baccalaureate nursing students in Korea.

**Methods:**

The participants were 99 baccalaureate nursing students. Content validity using content validity index (CVI), construct validity using exploratory factor analysis (EFA) and confirmatory factor analysis (CFA), and internal consistency using Cronbach’s alpha coefficient were assessed.

**Results:**

Both Item-CVIs and Scale-CVI were acceptable. EFA supported the unidimensional latent structure of Korean DASH-SV and results of CFA indicated 6 items converged within the extracted factor, significantly contributing to the factor (*p* ≤ .05). Items were internally consistent (Cronbach’s α = 0.82).

**Conclusion:**

The Korean version of the DASH-SV is arguably a valid and reliable measure of instructor behaviors that could improve faculty debriefing and student learning in the long term.

## Background

The vulnerabilities of relying on clinical training placements alone for new nurse readiness and the importance of nursing faculty being prepared to use alternate training modalities such as simulation have been studied [3]. Simulation-based learning is a potent complement to clinical placements and, in some countries, a potent substitute up to a certain curriculum proportion [1, 12]. However, this substitution requires a trained faculty cadre who are comfortable with simulation-based learning including debriefing. The challenge in many Korean nursing programs is that there is little or no faculty development for crucial debriefing skills [20].

One approach to closing this gap is to utilize debriefing assessment tools to create standards and develop shared agreements among faculty as to what constitutes high-impact and psychologically safe debriefing. Inviting student assessment of faculty debriefing skills leverages the ubiquitous presence of students to provide feedback via ratings. To date, to the authors’ knowledge, there is no such debriefing tool widely available and validated in the Korean language. To address this need, our team translated and conducted several tests of the validity of a Korean language translation of a leading debriefing assessment tool, the Debriefing Assessment for Simulation in Healthcare (DASH).

The DASH tool allows for the evaluation of instructor behaviors that facilitate learning and change in experiential contexts based on six elements, each scored on a 7-point scale with 1 = extremely ineffective/detrimental to 7 = extremely effective/outstanding [2, 21]. There are three ways in which instructors can seek evaluation: self-evaluate (Instructor Version) or receive evaluation from students (Student Version) or trained raters (Rater Version). Instructors, students, or raters can each use a short or long form to provide ratings and feedback on six DASH elements or twenty-three behaviors associated with the elements. While these tools, founded on identical elements and behaviors, are available in multiple languages, a version that has been formally translated into Korean and tested for psychometrics is not yet available. Thus, the purpose of the study was to develop a Korean version of the DASH-Student Version (SV), which can be used to translate all other versions and test its reliability and validity among baccalaureate nursing students in South Korea.

## Methods

### Design

**Fig. 1:**
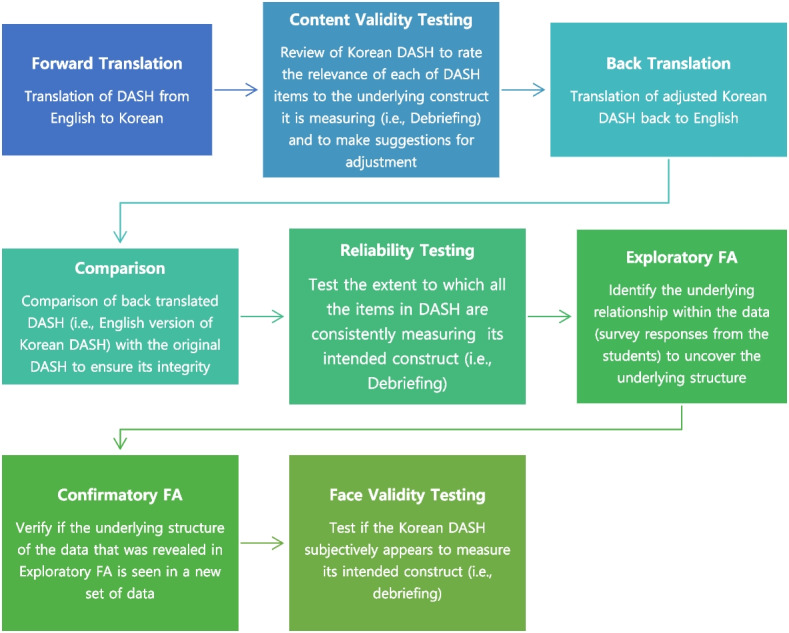
An outline of Translation and Psychometric Testing Process. *Note. DASH: Debriefing Assessment for Simulation in Healthcare; FA: Factor Analysis;*

The World Health Organization’s process of translation and adaptation of the instrument was used to develop a Korean version of the DASH-Student Version [23]. Psychometric measurement aspects of the Korean version of the DASH were tested for its validity and reliability using data collected from a survey. Figure 1 outlines the translation and psychometric testing process.

### Setting and participants

A convenience sample of nursing students was recruited from one college of nursing in a major university in a large metropolitan city in South Korea. Participants who were eligible were (1) 4th year nursing students who have experienced both simulation education and clinical experiences, and (2) who were currently taking an “Integrated comprehensive clinical simulation” course that included various simulation scenarios that covered clinical subject matter including medical-surgical, pediatric, gerontology and emergency nursing. Subjects that were learned throughout the entire program. This simulation course provided students with an opportunity to apply what they had learned from previous courses and demonstrate their readiness for clinical practice as senior students.

**Fig. 2:**
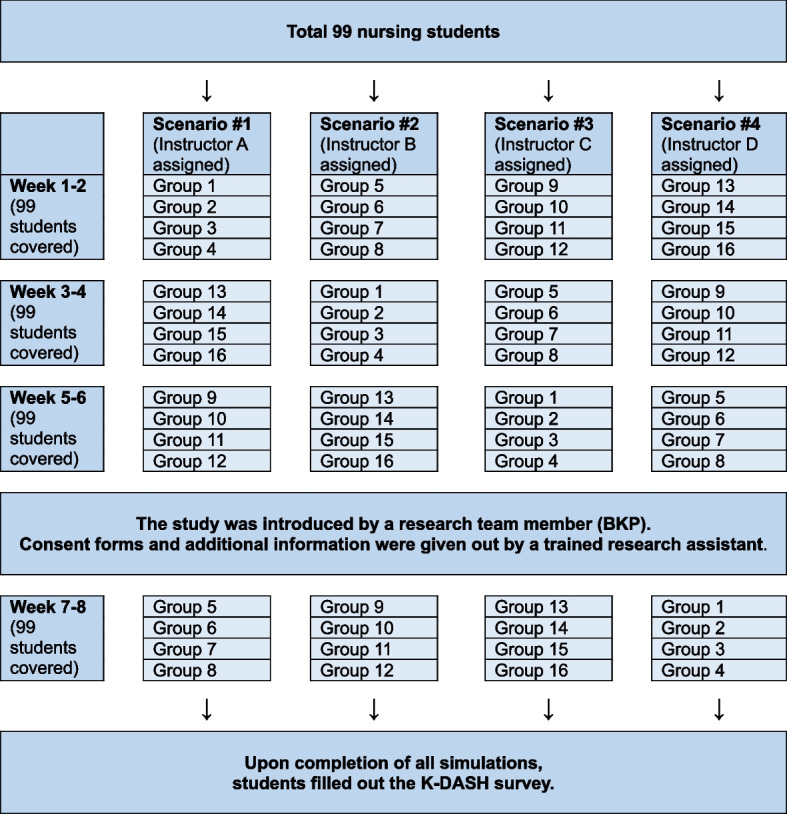
Flow of the simulation offerings and data collection. *Note. Each group had 6–7 students*

The four simulation scenarios that this cohort of students participated in were (1) a diabetic patient experiencing hypoglycemia, (2) a pediatric patient with febrile seizure, (3) an older adult patient at risk for falls, and (4) a post-operative orthopedic patient. Simulation and debriefing of each of the cases were facilitated by an instructor as groups of 6–7 students rotated through all four cases as a part of their regular simulation experience, over a period of 8 weeks (Fig. 2). Recruitment and data collection were conducted at the end of the fourth scenario for students to grow comfortable with simulation and debriefing, and to control the number of simulation and debriefing exposure.

### Translation

The Korean version of the DASH was developed following the process of translation and adaptation of the instrument proposed by the World Health Organization [23]. First, we acquired permission from the original developers of DASH [2]. Second, one bilingual professional in nursing who is experienced in facilitating clinical simulations independently conducted forward translation. Third, an expert panel, consisting of six faculty members of nursing schools currently teaching clinical simulation courses, evaluated the translated instrument for the content validity index (CVI). The expert panel rated each item of the K-DASH in terms of its relevance to the underlying construct on a 4-point scale (1 = not relevant, 2 = somewhat relevant, 3 = quite relevant, 4 = highly relevant) [6]. In addition, the expert panel commented on each item if they had any suggestions or questions. Through the expert panel discussion, the translated instrument was adjusted, and the K-DASH was produced for back-translation. Then, the back-translation of the K-DASH from Korean to English was conducted by a bilingual translator. The back-translated K-DASH (in English) was compared against the original DASH by one of the original developers of DASH-Student Version (R.S.). Feedback was incorporated to yield the final K-DASH, which was used for psychometric testing at a college of nursing in a major university in a large metropolitan city in South Korea.

### Data collection

Data were collected using a self-reported questionnaire at the end of the fourth case. To recruit study participants prior to the fourth simulation scenario, a researcher [BKP] provided the overall information of this study including that participation in this study would not affect their grade in this course. Then, the researcher left the classroom to eliminate the risk of unintended coercion by faculty. A trained research assistant provided additional explanation and distributed consent forms to students who volunteered to participate in the study. After the simulation and debriefing were complete, participants filled out the K-DASH survey which also included an opportunity to provide feedback on the K-DASH items themselves (e.g., related to readability of the items). Figure 2 shows the flow of simulation offerings and data collection. Students’ comments were taken into consideration in finalizing all six versions of the K-DASH, i.e., short and long DASH instructor, rater, and student versions.

### Ethical considerations

This study was reviewed and approved by the corresponding author’s Institutional Review Board (2018–0050). The researchers did not engage in recruitment or data collection. The informed consent was voluntarily obtained from all participants. Research participants were provided with $10 worth of stationery as a token of appreciation, as disclosed in the consent form.

### Data analysis

Data were first entered into IBM SPSS 25.0 (IBM Corp, Armonk, NY, USA) for data cleaning and analyzed in both IBM SPSS and Mplus 8.1 (Muthén & Muthén, Los Angeles, CA, USA). Descriptive statistics were used to characterize the sample. Inter-item correlations were computed to evaluate the adequacy of items and Cronbach’s alpha was used to measure reliability.

To determine the content validity index for individual items (I-CVI), six members of the expert panel rated each K-DASH item in terms of its relevance to the underlying construct on a 4-point scale. All members of the expert panel had more than 3 years of simulation and debriefing experience. Then, I-CVI was computed for each item as the number of experts giving a rating of either 3 or 4, divided by the number of experts which led to the proportion of agreement about relevance. The content validity index for the scale (S-CVI) was calculated as the average of the I-CVIs for all items on the scale [19]. An I-CVI higher than 0.78 is considered excellent, and S-CVI higher than 0.80 is considered acceptable (Polit and Beck, 2020).

While the items in the K-DASH student version were measured on a 7-point scale, most subjects responded positively on all items between 4 and 7, with higher scores indicating positivity. Using the first group (*n* = 49) exploratory factor analysis (EFA) using polychoric correlation coefficients was performed to examine the underlying theoretical structure of the translated K-DASH-Student Version; Cronbach’s alpha was computed for test reliability. Lastly, confirmatory factor analysis (CFA) with a diagonally weighted least square (WLSMV) estimator rather than a maximum likelihood (ML) estimator was performed to evaluate the factorial validity of the K-DASH student version to compare with the second group (*n* = 50). For the CFA, we used Kenny’s recommendations [14] for a good fitting model of (a) the ratio of chi-square to degrees of freedom (df should be 2:1 or less with non-significance; (b the root mean square error of approximation (RMSEA should be 0.08 or less; (c the confirmatory fit index (CFI and Tucker-Lewis index (TLI should be 0.95 or greater; (d the standardized Root Mean Square Residual (SRMR should be 0.08 or less. All statistical significances were reported at *p* ≤ 0.05.

### Face validity and finalization of K-DASH

Based on the statistical analysis and comments from students, the K-DASH was adjusted. The expert panel that completed the CVI reviewed the updated K-DASH for face validity and subjectively evaluated if the tool is viewed as achieving its intended goals of assessment of debriefing.

## Results

All 99 students who were eligible participated and completed the survey. Most of the students who completed the survey were female (84.8%). On the 7-point DASH scale (from 1 to 7), the average score of 6 items in the Korean version of the DASH was 6.12 (range 5.98–6.27, SD 0.803) (Table 1). The frequency of students’ responses to each item was mainly distributed between 4 and 6 without any from 1 through 3.

**Table 1:** Total and item means for the Korean version of the DASH-SV (*N* = 99)

Korean DASH-SV Elements (7-point Likert Scale)	Mean ± SD
1. The instructor set the stage for an engaging learning experience	6.06 ± .767
2. The instructor maintained an engaging context for learning	6.11 ± .698
3. The instructor structured the debriefing in an organized way	6.19 ± .710
4. The instructor provoked in-depth discussions that led me to reflect on my performance	6.10 ± .863
5. The instructor identified what I did well or poorly—and why	6.27 ± .843
6. The instructor helped me see how to improve or how to sustain good performance	5.98 ± .937
Total	6.12 ± .803

### Inter-item correlations and reliability

Inter-item correlations were computed for items in the Korean DASH to examine the adequacy of items. All 6 items were significantly correlated with each other with coefficients ranging between 0.29 and 0.62, on or above the recommended value of *r* = 0.30 (*p* ≤ 0.05). The Cronbach’s alpha was 0.82 suggesting that the items have relatively high internal consistency.

### Content validity

The content validity index for the scale (S-CVI) was calculated as the average of the I-CVIs for all items on the scale [19]. An I-CVI higher than 0.78 is considered excellent, and S-CVI higher than 0.80 is considered acceptable (Polit and Beck, 2020). Both the I-CVI and S-CVI of the Korean version of the DASH were excellent with I-CVI higher than 0.83 for all items and the S-CVI of 0.94 (Table 2), suggesting that K-DASH is measuring its underlying construct of debriefing. The overall recommendations from the expert panel were not on the content but rather, focused on conserving the original meaning of DASH items that could sometimes be altered in the process of translation from one language to another and back again. For example, “set the stage for” in Korean does not deliver the full sense of the original meaning, thus we translated this phrase as “prepare the conditions for.” The authors made every effort to develop a Korean version that accounts for the simulation education environment and culture in Korea while conserving the original intent and underlying meaning of each of the behaviors and elements.

**Table 2:** Individual content validity index (CVI) and scale CVI scores for the Korean version of the DASH-SV

Korean DASH-SV Elements	Expert 1	Expert 2	Expert 3	Expert 4	Expert 5	Expert 6	Individual CVI
1. The instructor set the stage for an engaging learning experience	3	3	3	3	4	4	1.00
2. The instructor maintained an engaging context for learning	3	3	4	4	4	4	1.00
3. The instructor structured the debriefing in an organized way	3	4	3	4	4	4	1.00
4. The instructor provoked in-depth discussions that led me to reflect on my performance	3	3	2	4	3	4	0.83
5. The instructor identified what I did well or poorly—and why	4	4	4	4	4	4	1.00
6. The instructor helped me see how to improve or how to sustain good performance	2	4	4	3	4	3	0.83
Scale CVI							0.94

### Construct validity

#### Exploratory factor analysis

Structured debriefing using Kaiser’s rule [13] of eigenvalue greater than 1 to extract factors and ProMax rotation produced a one-factor solution with an eigenvalue greater than 1, accounting for 55.4% of the variance. This suggested a unidimensional latent structure of the Korean version of the DASH that, based on the data collected from students K-DASH, measures one construct (i.e., debriefing).

#### Confirmatory factor analysis

Results of confirmatory factor analysis with the WLSMV estimator indicated that the fit indices were satisfactory with a nonsignificant χ^2^/df ratio of 1.39, RMSEA of 0.089, SRMR of 0.065, CFI of 0.98, and TLI of 0.97. Additionally, all standardized loadings for items were statistically significant (*p* ≤ 0.05) with moderately large values ranging between 0.56 and 0.90, indicating that each of the 6 items converges within the extracted factor and significantly contributes to the factor. The composite reliability (CR) was 0.87 and the average of variance extracted (AVE) was 0.53, indicating good convergent validity, the degree of confidence that a trait is well measured by its indicators [9]. These results support the findings of EFA and the reliability of the scale, suggesting that the set of items in K-DASH relates to the given latent variable (i.e. instructor behaviors that facilitate learning and change in experiential contexts) and captures a good amount of the variance in the trait or the latent variable. The results of both EFA and CFA are summarized in Table 3.

**Table 3:** Reliability and validity of the Korean version of the DASH-SV

Items	Factor loadings (EFA)	Std loadings (CFA)	Cronbach’s alpha	CR	AVE
1	.77	.771**	.82	.87	.53
2	.67	.74**			
3	.74	.73**			
4	.80	.56**			
5	.71	.66**			
6	.78	.90**			

^**^p value < 0.001; *CR* composite reliability, *AVE* average of variance extracted

## Discussion

The internal consistency of the Korean version of the DASH was high (Cronbach’s alpha = 0.82), comparable to that of the original instrument (Cronbach’s alpha = 0.89), although the original study was conducted using the rater version [2]. This finding aligned with the reliability reported for a study conducted in the USA, using the student version with nursing students (Cronbach’s alpha = 0.82) [7]. The results of EFA suggested a unidimensional latent structure of the Korean version of the DASH, and the results of CFA confirmed that each of the 6 items related to or converged with the extracted factor. Therefore, this suggests that the 6 items captured a good amount of the instructor debriefing behaviors which the K-DASH is intended to assess. To the best of the authors’ knowledge, this is the first study exploring the content validity and construct validity specifically using CVI, EFA, and CFA and may serve as a comparison metrics for future psychometric studies.

In South Korea, the use of simulation in nursing colleges is growing sharply, but debriefing methods are often neglected by instructors [11]. There is a lack of simulation instructors who are trained for structured debriefing [20]. The largest gap between the significance of practice and actual performance was reported to be in reflection and facilitation [20]. Structured debriefing is one of the important characteristics of effective debriefing in simulation-based learning [8]. From the students’ perspective, the debriefing process from addressing emotions to reflecting and summarizing, improved their learning [8]. To provide structured debriefing, facilitators require formal training in debriefing techniques [22] as well as formal tools, such as the DASH, to assess debriefing skills. Considering the current situation of nursing simulation in Korea, the use of the DASH would be beneficial for novice debriefers with less or no formal training in debriefing structure and techniques because it will provide a reference for the standard behaviors required for effective debriefing, organized into six key elements [2]. The DASH also suggests the need to address physical or environmental barriers to quality debriefing, such as lack of time and space for debriefing or the large number of students in a simulation session that are identified as reasons why debriefing is overlooked or shortened in South Korea [11]. The Korean version of the DASH can help instructors improve the effectiveness of their debriefings and thereby contribute to promoting the quality of nursing simulation education in Korea.

Of note, negative answer choices: (1) extremely ineffective/detrimental, (2) consistently ineffective/very poor, or (3) mostly ineffective/poor—were not selected by the student participants in this study. Ratings were primarily distributed between 4 through 6 without any from 1 through 3. The total mean score was 6.12 out of 7 (extremely effective/outstanding) and each element's mean score ranged from 5.98 to 6.27. This result was different from a study conducted in the US and Australia with students, doctors, and registered nurses, which had a wider range of DASH mean scores from 5.00 to 7.00 [5]. Another study with pediatric and anesthesia residents simulation education reported a wide range of DASH-Student Version median scores from 4.00 to 7.00 [10]. While these results may purely reflect the quality of the debriefing, it would be prudent to consider the cultural aspects. In certain Asian cultures, students are expected to show respect to teachers,often students do not consider themselves to be in a position to negatively critique their professors. One of the students commented that it would not be appropriate to use those strong negative descriptions, such as detrimental, in their ratings. Some students seemed to be comfortable with giving a rating of 7 (strong positive descriptions. Considering students' tendency to choose the neutral mid-point category, a 7-point rating scale may provide a wider range of variance to differentiate raters’ perspectives, compared to a 5-point rating scale. Conversely, reducing the number of choices to a 5-point rating scale may yield meaningful differences (e.g., a difference between 4 and 5 could be greater in a 5-point rating scale than a 7-point rating scale). Another cultural difference was the participation rate of students 100% of eligible students participated even when a research assistant conducted recruitment and data collection instead of their professor. In Korea, students are generally accepting of academic assignments and research. It is not uncommon to see exceptionally high participation rates [15, 18]. Regardless of cultural differences, the high participation rate could be attributed to the fact that students completed K-DASH right after debriefing.

The final version of the K-DASH, evaluated for face validity by the expert panel, can be found (https://harvardmedsim.org/debriefing-assessment-for-simulation-in-healthcare-dash-korean).

## Limitations

As with all psychometric testing studies, the findings from this particular study are based on data collected in this setting and population. Further psychometric testing in different Korean settings with different populations is warranted to increase generalizability. Additionally, future investigation with a larger sample size would confirm the stability of results in this study as the current study had a minimal sample size needed to split the sample for both EFA and CFA. The split was necessary to conduct EFA and CFA on a different sample as running the analysis on the same sample can introduce bias, leading to overfitting and compromising the validity of findings. While a larger sample size should be preferred to ensure the stability and reliability of the factor structure, studies with smaller sample sizes can still provide meaningful insights under certain circumstances. As MacCallum et al. [16] demonstrated, for example, the factor recovery can be acceptable with smaller samples if the communalities are high and factors are well-defined, which was the case for K-DASH containing 6 items designed to form a single factor. Additionally, item factor loadings for this study were substantial and significant, providing meaningful preliminary insight about the factor structure of K-DASH.

While the translation was conducted following the process of translation and adaptation of the instrument proposed by the World Health Organization and was validated by content experts, linguistic and cultural nuances interpreted could vary by learners in different age groups (e.g., baby boomers and generation z students), settings (e.g., academic or healthcare industry), disciplines (including intra and inter-disciplinary), or with different life experiences (e.g., those with or without study abroad experiences). Considering that “learning assumptions”, cultural context, and team dynamics may influence the active reflection and participation required in a debriefing session, the culturally sensitive application of models and tools is crucial for maximized learning outcomes [4]. This translated tool can benefit from further linguistic/cultural validation following the process used by Muller-Botti and colleagues [17].

Cultural differences, such as preference towards positive rating versus negative rating can be a limitation. Providing students with an orientation to the tool, including an introduction to the purpose of the assessment and the concept of constructive feedback may help address this tendency.

## Conclusions

As nursing schools worldwide scramble to meet the growing demand for work-ready graduates, robust faculty development is crucial. One proven way to prepare faculty to lead robust simulation-based learning activities is to strengthen their debriefing skills via standards and feedback. The Korean version of the DASH appears to be a reasonably valid and reliable measure of instructor behaviors that facilitate learning and change in faculty skills, and by extension, nurse trainee skills in experiential contexts in Korea.

## Data Availability

The datasets used and/or analyzed in this study are available from the corresponding author on reasonable request.

## Abbreviations

DASH^©^: The Debriefing Assessment for Simulation in Healthcare
DASH-SV: DASH-Student Version
CVI: Content validity index
EFA: Exploratory factor analysis
CFA: Confirmatory factor analysis
WLSMV: With diagonally weighted least square
ML: Maximum likelihood
RMSEA: Root mean square error of approximation
CFI: Confirmatory fit index
TLI: Tucker-Lewis index
SRMR: Standardized root mean square residual
CR: Composite reliability
AVE: Average of variance extracted
